# Knock-down of AHCY and depletion of adenosine induces DNA damage and cell cycle arrest

**DOI:** 10.1038/s41598-018-32356-8

**Published:** 2018-09-18

**Authors:** Lucija Belužić, Ivana Grbeša, Robert Belužić, Jong Hoon Park, Hyun Kyung Kong, Nevenka Kopjar, Guadalupe Espadas, Eduard Sabidó, Adriana Lepur, Filip Rokić, Ivanka Jerić, Lidija Brkljačić, Oliver Vugrek

**Affiliations:** 1 Ruđer Bošković Institute, Laboratory for Advanced Genomics, 10000 Zagreb, Croatia; 2 Bar-Ilan University, Cellular and Developmental Biology, Ramat-Gan, 5290002 Israel; 3 Sookmyung Women’s University, Department of Biological Science, Seoul, 140-742 Korea; 4 Institute for Medical Research and Occupational Health, Mutagenesis unit, 10000 Zagreb, Croatia; 5 Proteomics Unit, Centre de Regulació Genòmica (CRG), Barcelona Institute of Science and Technology (BIST), Dr. Aiguader 88, 08003 Barcelona, Spain; 6 Universitat Pompeu Fabra (UPF), Dr. Aiguader 80, 08003 Barcelona, Spain; 7 BIOCenter, Microbiology Laboratory, 10000 Zagreb, Croatia; 8 Ruđer Bošković Institute, Division of Organic Chemistry and Biochemistry, 10000 Zagreb, Croatia

## Abstract

Recently, functional connections between S-adenosylhomocysteine hydrolase (AHCY) activity and cancer have been reported. As the properties of AHCY include the hydrolysis of S-adenosylhomocysteine and maintenance of the cellular methylation potential, the connection between AHCY and cancer is not obvious. The mechanisms by which AHCY influences the cell cycle or cell proliferation have not yet been confirmed. To elucidate AHCY-driven cancer-specific mechanisms, we pursued a multi-omics approach to investigate the effect of AHCY-knockdown on hepatocellular carcinoma cells. Here, we show that reduced AHCY activity causes adenosine depletion with activation of the DNA damage response (DDR), leading to cell cycle arrest, a decreased proliferation rate and DNA damage. The underlying mechanism behind these effects might be applicable to cancer types that have either significant levels of endogenous AHCY and/or are dependent on high concentrations of adenosine in their microenvironments. Thus, adenosine monitoring might be used as a preventive measure in liver disease, whereas induced adenosine depletion might be the desired approach for provoking the DDR in diagnosed cancer, thus opening new avenues for targeted therapy. Additionally, including AHCY in mutational screens as a potential risk factor may be a beneficial preventive measure.

## Introduction

S-adenosylhomocysteine hydrolase (AHCY; SAHH) catalyses the hydrolysis of S-adenosylhomocysteine (SAH) to adenosine (Ado) and homocysteine (Hyc) in living organisms^1^. SAH is both a leftover metabolite of cellular transmethylation reactions and a strong competitive inhibitor of methyltransferases^2^. Proper activity of AHCY is essential for maintaining the cellular methylation potential, which is determined by the ratio of the S-adenosylhomocysteine (SAH) and S-Adenosylmethionine (SAM) metabolites^3,4^. The importance of rapid removal of SAH by AHCY has been underscored by the discovery of AHCY deficiency in humans^5^. AHCY deficiency is a rare and potentially lethal multisystem disorder^6,7^ of methionine metabolism caused by the reduction of AHCY enzymatic capabilities as a result of allelic mutations in the coding region of the *AHCY* gene^8–11^.

Recently, several studies noted the connections between AHCY and cancer from various standpoints: as a player that possibly regulates the cancer phenotype^12–14^, as a druggable candidate^15^, or as a promising biomarker^16–19^. Based on these reports, the involvement of AHCY in the molecular mechanisms of cancer is undisputable. Recently, AHCY-driven mechanisms have been discussed, such as the treatment of liver carcinoma cells (HepG2) with AHCY inhibitors, where the DNA damage response is predicted to be enhanced by endogenous genotoxicity due to DNA damage and subsequent perturbation of the cellular epigenome^20^; however, the mechanisms by which AHCY affects cancer are still elusive. Additionally, in regard to research on HepG2, most studies evaluated the genotoxicity of many direct and indirect mutagens and compounds with unknown or poorly known mechanisms of action^21–24^, thus leaving many questions unanswered.

It is worthwhile to emphasize, though, that depending on the cancer type studied, the AHCY levels may have notably different effects on the cell phenotype. Reducing AHCY activity causes the invasive ability of breast cancer and glioblastoma cell lines to diminish^12,13^, while the elevation of AHCY activity in oesophageal squamous cell carcinoma causes apoptosis and inhibition of cell migration and adhesion without causing changes in cell proliferation or the cell cycle^14^.

AHCY deficiency has been implicated in hepatic pathology of AHCY during the past decade^25^, and a recently reported case of hepatocellular carcinoma in an adult^26^ allowed us to examine the role of AHCY and its mechanism of action in the cell cycle, cellular proliferation and the DNA damage response in a suitable cell line, such as HepG2. Additionally, despite the well-described metabolomic parameters in previous research on AHCY deficiency, one question remains unsolved: What are the implications of adenosine, the primary product of AHCY hydrolytic activity, but not homocysteine, on the cellular metabolism?

Certainly, connections between adenosine and cancer have been established, showing stimulative effects on cancer cell proliferation^27,28^ and other important roles in inflammation or immunity. However, current research is mainly focused on extracellular adenosine, whereas increased intracellular adenosine concentrations seem to facilitate the development and sustainability of an immunosuppressed cancer microenvironment and contribute to angiogenesis and metastasis^29^. Additionally, hydroxyurea (HU) treatment in cancer-related studies showed a connection between dNTP levels^30^, demonstrating the importance of dATP as a major contributor in the proper progression of DNA replication.

Thus, to shed light on AHCY, adenosine and other intracellular processes, we pursued a multi-omics approach in combination with basic molecular and cellular biology procedures and provided extensive and strong evidence that adenosine depletion is involved in cell cycle arrest, decreased cellular proliferation, and DNA damage induction. Further, we propose a mechanism based on adenosine depletion that can explain both the pathology in the latest case of AHCY deficiency^26^, where mild inactivation of AHCY activity causes the late-onset of typical disease symptoms, and the path to the development of hepatocellular carcinoma as a result of AHCY dysfunction.

## Results

### AHCY knockdown impacts cellular methylation potential, cell morphology, cell cycle and proliferation rates

Recent studies have indicated a functional connection between cancer and S-adenosylhomocysteine hydrolase (AHCY) activity, particularly in liver cancer^26^. To shed light on the crosstalk between AHCY and cancer, we opted for a multi-omics approach using hepatocellular carcinoma cell lines that show high expression levels of AHCY^31^, making them a suitable model system.

**Figure 1:**
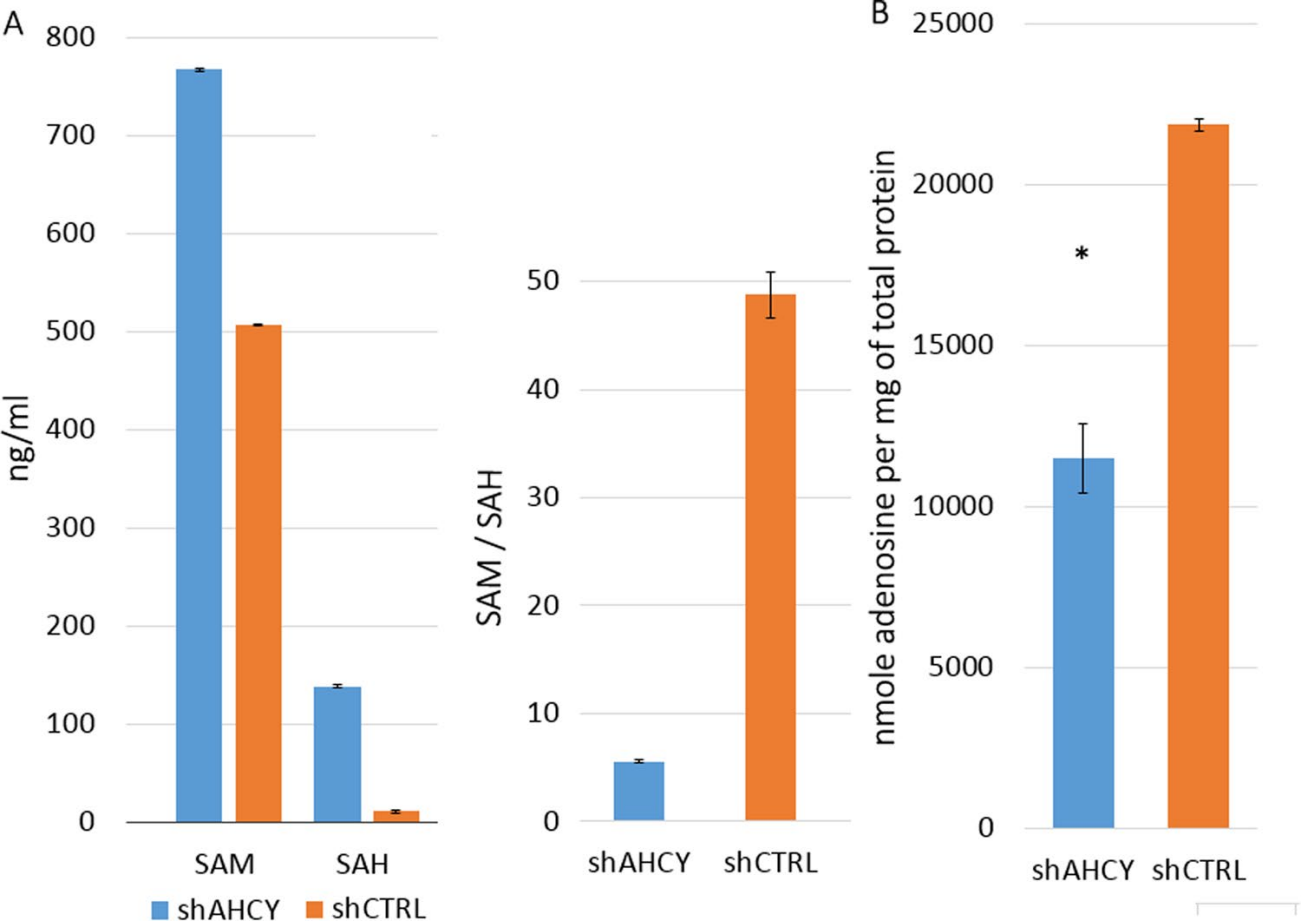
SAM/SAH and adenosine measurements. (**A**) Levels of SAM and SAH (ng/ml) and their ratio (SAM/SAH) in the lysates of AHCY-silenced and control cells, as measured by LC-MS/MS. ± SD is represented as vertical line and is based on three independent measurements. (**B**) Adenosine was determined in the deproteinised cell lysates of AHCY-knockdown and control cells using an Adenosine Assay kit (BioVision) and represented as nmole adenosine per mg of total protein in the cell lysate. Vertical lines represent ± SD of two 2 individual measurements performed in triplicates for each sample (*means P < 0.05; determined by two tailed t-test).

By measuring the concentrations of S-adenosylmethionine (SAM) and S-adenosylhomocysteine (SAH) in cell lysates, we assessed the impact of AHCY silencing on the cellular methylation potential of HepG2 cells. The elevation of these two metabolites is the main indication of AHCY deficiency^5^ and serves as a valuable reference for evaluating the efficiency of AHCY knockdown. Both SAM and SAH were found to be highly elevated in the lysates of shAHCY cells (Fig. 1A), thereby dramatically shifting the SAM/SAH ratio in HepG2 from approximately 48 in control cells to 5 in silenced cells (Fig. 1B). In comparison, the SAM/SAH ratio in human plasma is approximately 4 to 1 in favour of SAM, whereas patients suffering from AHCY deficiency show a reversal in the SAM/SAH ratio of 2 to 1 in favour of SAH^5^. As HepG2 is metabolically highly active, a complete reversal in the SAM/SAH ratio, as observed in patients, is not likely, although there is a clear tendency for the SAM/SAH ratio to shift.

**Figure 2:**
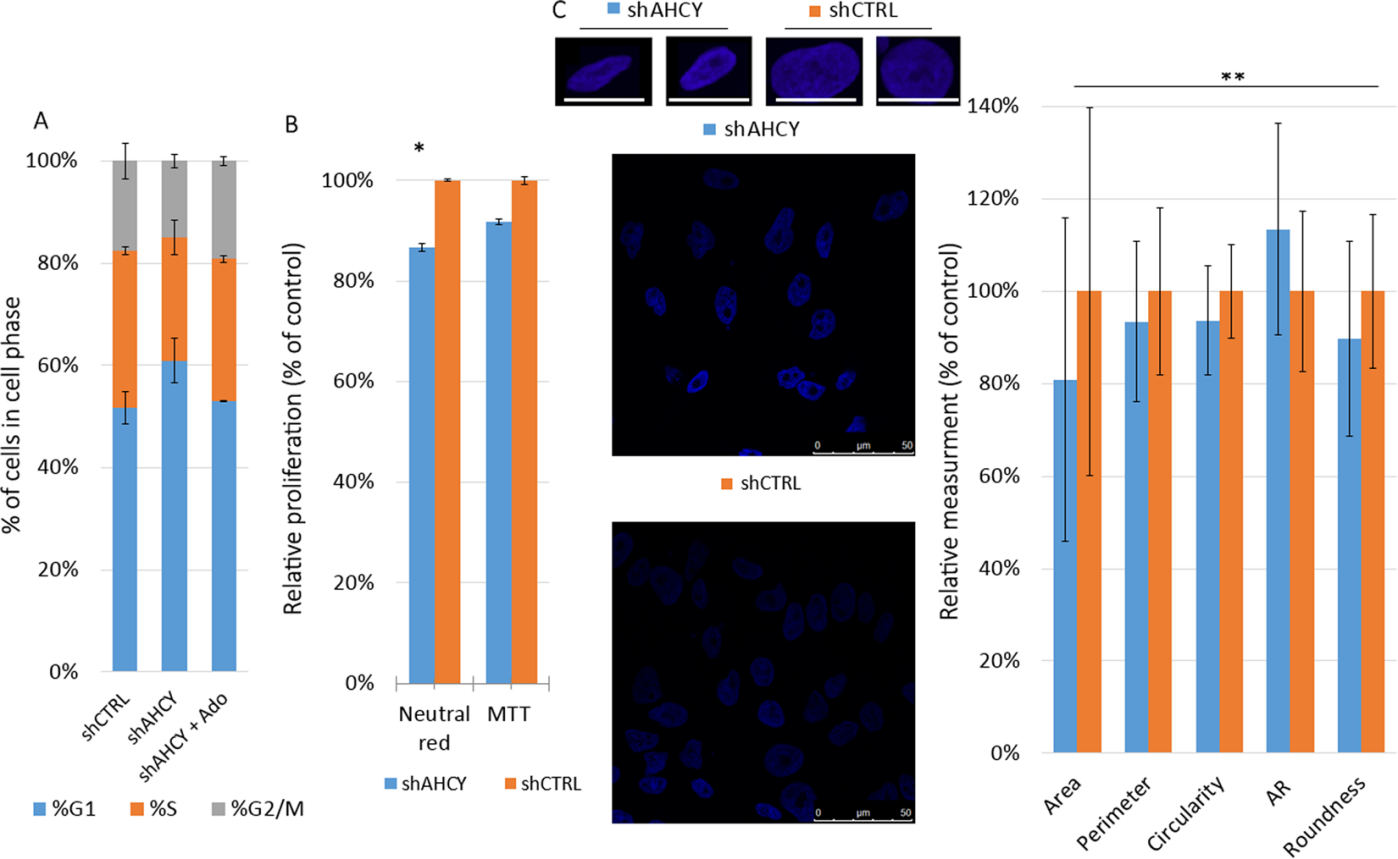
Morphometric and cell cycle analysis. (**A**) Effects of AHCY silencing on the cell cycle distribution determined by flow cytometry after staining with propidium iodide. G1, S and G2/M indicate the cell phase in question. The graphs show the mean ± SD of three independent experiments performed in triplicate for each tested sample. (**B**) Changes in the cell proliferation rates of AHCY-silenced cells compared to control cells, as determined by NRA and MTT tests. The graphs show the mean ± SD of three independent experiments performed in at least 10 wells/cell line (* means P < 0.0.05; determined by two tailed t-test). (**C**) Upper panel: representative nuclei images (blue: DAPI). Centered is a field of view containing >10 cells for each condition. Graph: changes in the main nuclear morphometric features in % when compared with shCTRL set as 100% (mean of more than 100 nuclei; ** means p < 0.01 determined by two tailed t-test). Imaged with a Leica SP8 X FLIM confocal microscope (HC PL APO CS2 63 ×/1.40 OIL objective); scale bar, 50 µm.

The effect of AHCY silencing on the main nuclear morphometric features and form factors can be seen in the graphical representation in Fig. 2C. A total of 100 nuclei per cell line were studied, and significant changes were observed in the following categories for shAHCY cells: a decrease in cell area, perimeter, circularity, and roundness and an increase of the AR form factor (see Confocal microscopy and image analysis). Namely, silencing of AHCY expression results in the transformation of HepG2 nuclei to a smaller and more elliptical shape (Fig. 2A), meaning that they resemble normal cells rather than cancerous cells^32^.

Visible differences in the growth rate were noticed while passaging the shAHCY and shCTRL cells. Since the influence of lower AHCY activity on the mitochondria or lysosomal stability has not been investigated, a possible bias was excluded by using both MTT and neutral red assays to evaluate cell proliferation rates. We noticed 8.4% decreases in cell proliferation rates with the MTT assay and 13.4% decreases with the neutral red test (Fig. 2B), which indicate that both assays are appropriate for studying the effects of AHCY silencing on the proliferation of cells, therefore confirming the observed phenotype.

We then assessed the cell cycle distribution of shAHCY cells by flow cytometry. DNA staining with PI revealed the accumulation of cells in G1 phase from 50.90% in shCTRL cells to 59.83% in shAHCY cells (Fig. 2A), with a corresponding decrease of shAHCY cells in the S and G2/M phases. Thus, it can be concluded that the pathways causing G1/S checkpoint arrest are main contributors to cell cycle changes in Hep G2 cells after AHCY silencing.

**Figure 3:**
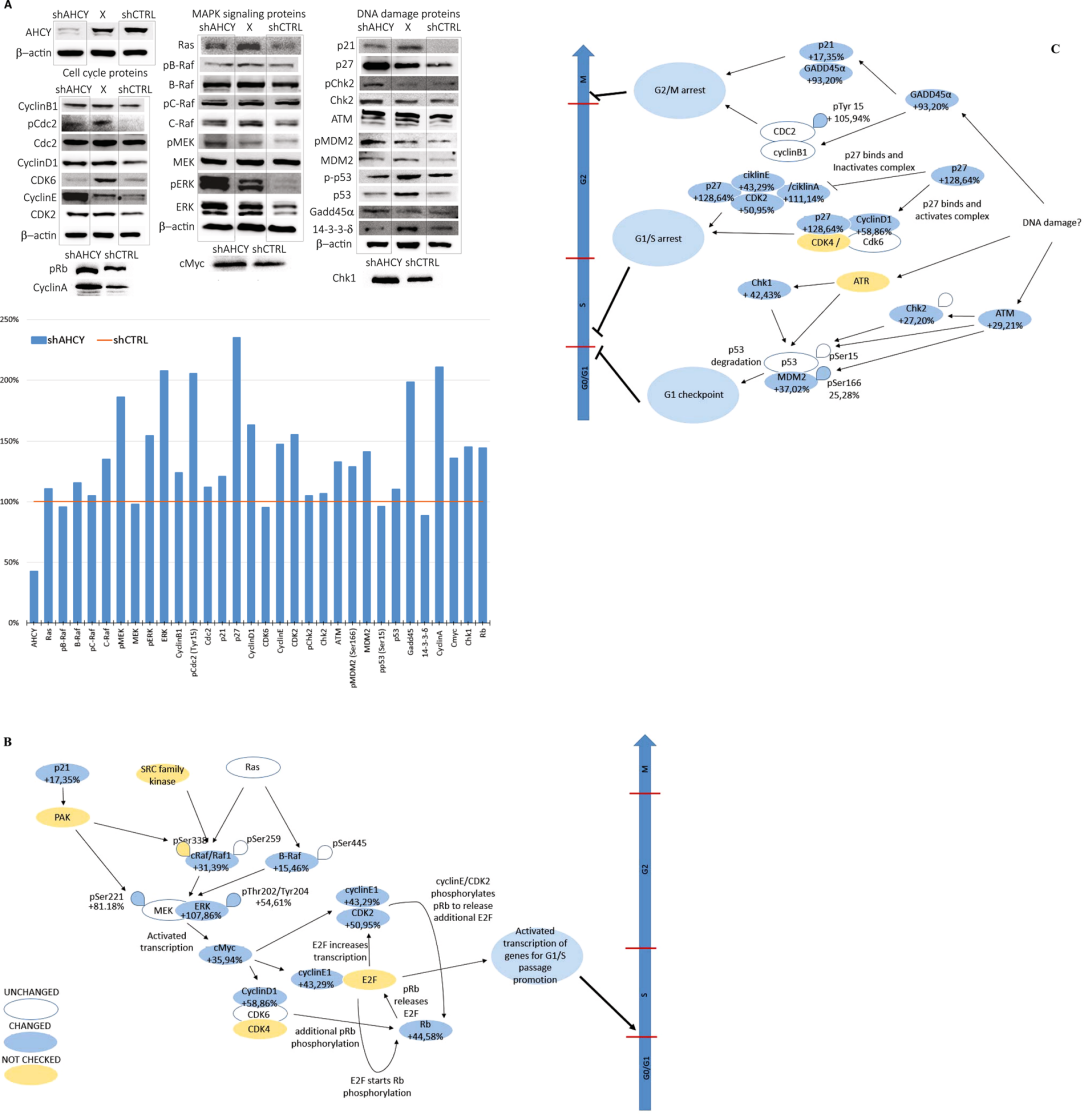
Proposed effects of AHCY silencing on cell cycle regulating proteins and checkpoints based on Western blotting results. (**A**) Upper panel: Protein expression levels (names listed in Table 2) analysed by Western blotting (30–80 μg of whole cell proteins loaded per well). Bands in lane marked with “X” were not analysed. Lower panel: Signal densitometry was performed using ImageJ software. Each band was normalized using β-actin as the loading control. The shAHCY signal for each protein was expressed as the % change versus shCRTL, which was set to 100% (orange line). (**B** and **C**) Schematic diagrams of the Western blot results, for which changes in the signal for each analysed protein in AHCY-silenced cell lysates are represented as the % change versus control cells (set as 100%). Signal densitometry was performed in ImageJ software, and each band was normalized using β-actin as the loading control. Maximum change between β-actin signals for the same sample on 10 membranes exposed at the same time is +/− 11.2% and was used to verify the degree of reproducibility of the method as well as to designate all the proteins with expression changes lower than +/− 11.2% as unchanged. “p” signifies the phosphorylated form of the protein. Arrows indicate the positive impact on the downstream molecule (activation), while bars represent the negative impact on the downstream molecule (repression). The large arrow indicates the cell cycle where the cell phases are marked with G1, S, G2 and M, while horizontal bars represent cell cycle checkpoints marked red if impacted by the proposed pathways.

Subsequently, analysing the levels of 29 proteins, including the phosphorylated versions (Fig. 3A) known to be involved in the cell cycle through various pathways and different upstream signalling pathways, allowed the assembly of a scheme with effects on the cell cycle checkpoints, as indicated in Fig. 3B,C. Protein levels were normalized using a β-actin loading control and compared with expression levels from the control cells.

**Figure 4:**
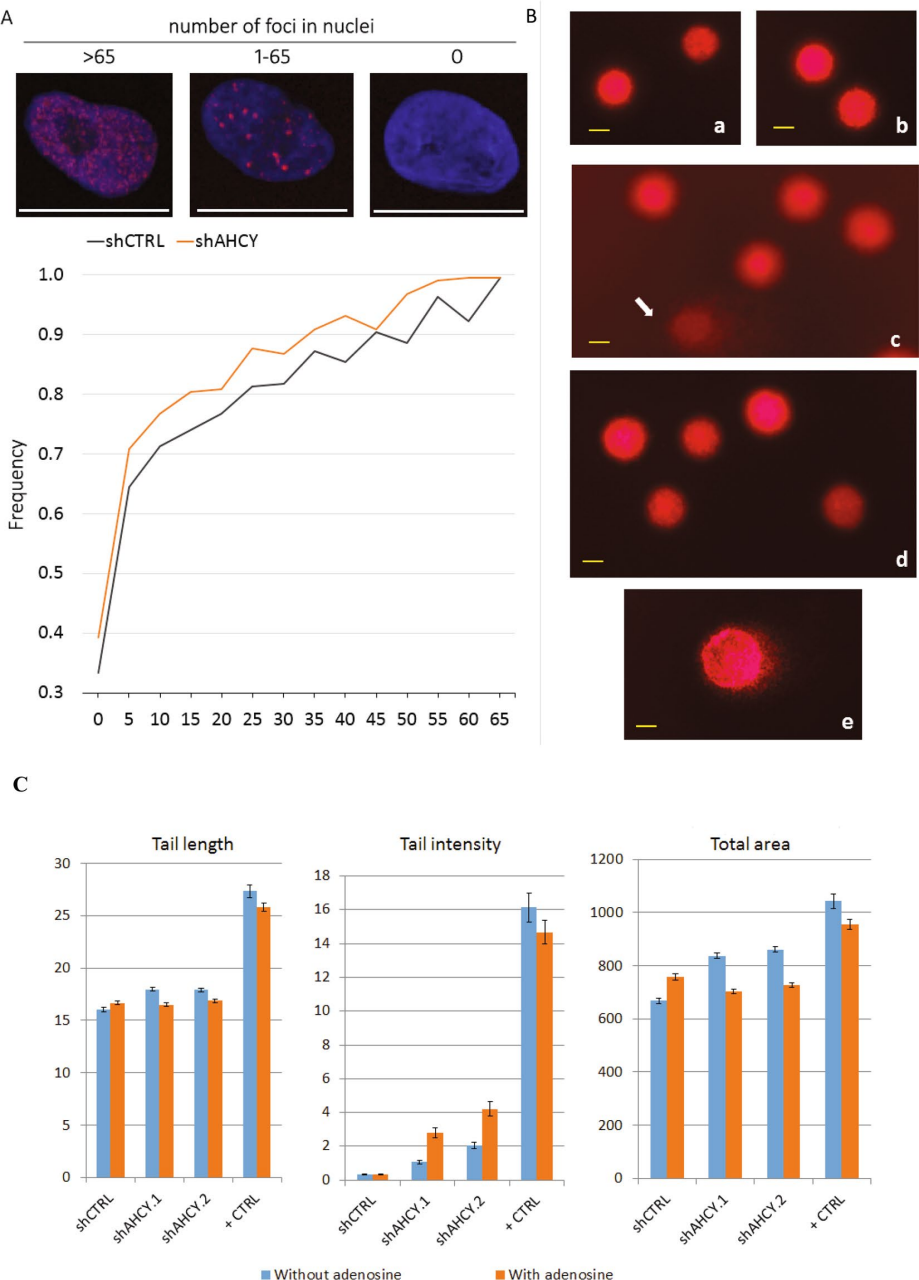
yH2AX spread analysis. (**A**) Upper panel: example of three types of cell nuclei after yH2AX immunocytochemistry regarding the number of foci (red: Alexa 594 γ-H2AX foci; blue: DAPI cell nucleus). Graph: frequency distribution of nuclei with 0 to 65 foci in increments of five (calculated from 220 nuclei per cell line; p = 0.00001 determined by two tailed t-test). Imaged with Leica SP8 X FLIM confocal microscopes (HC PL APO CS2 63 × /1.40 OIL objective). (**B**) Primary DNA damage in HepG2 cells estimated by the alkaline comet assay. Photomicrographs of the nuclei of HepG2 cells observed after the alkaline comet assay procedure. (a) control shCTRL cells; (b) control shCTRL cells cultivated with the addition of adenosine; (c) HepG2 cells with silenced ACHY (shAHCY), where an arrow indicates the damaged DNA that resembles comet-style features; (d) HepG2 cells with silenced ACHY (shAHCY) cultivated with the addition of adenosine. Agarose microgels were stained with ethidium bromide (20 µg/mL) and analysed under epifluorescence microscope (Olympus BX51), under 200× magnification. Photomicrographs acquired by the image analysis system Comet Assay IV^TM^ (Perceptive Instruments Ltd., UK). (**C**) indicators for DNA damage were (a) tail length, (b) tail intensity, and (c) total area. For each sample, three replicates were prepared, with 100 independent comet measurements per slide, with 300 measurements performed per sample. The image analysis system ‘Comet Assay IVTM’ (Perceptive Instruments Ltd., UK), in combination with an epifluorescence microscope (Olympus BX50, Japan) equipped with appropriate filters, under 200x magnification, was used for the analysis. The results are shown as the median/mean value, and the range of the measured values (min-max); scale bar, 20 µm. Statistical significance of the data was evaluated using descriptive statistics, ANOVA with post hoc Scheffé’s test (intra-group comparisons) and the Mann-Whitney U-test (inter-group comparisons). The level of statistical significance was set at P < 0.0.05. The abbreviations above the whiskers indicate which samples differ with statistical significance. For ANOVA, the abbreviations are as follows: nc – vs. corresponding negative control; # – vs. all other samples. A sign * designates the samples that showed a statistically significant increase of the studied comet parameter compared to the related clone with regard to the addition of adenosine. shAHCY.1 – cells with silenced AHCY, replica 1; shAHCY.2 – cells with silenced AHCY, replica 2., Positive control – shCTRL cells exposed *ex vivo* to 50 µM hydrogen peroxide for 10 minutes on ice. For each sample, three replicate slides were prepared.

The upper part of the scheme (Fig. 3B) combines all pathways that might be activated as cellular responses to various types of DNA damage. After a thorough analysis of the crucial players of cell cycle control pathways, we determined that G1/S arrest was likely caused by DNA damage. Although the protein levels of the ATR kinase, associated with single-stranded breaks, were not assessed, the protein levels of the downstream effector molecule Chk1 showed a significant increase. Nevertheless, it has been shown that DNA damage induced phosphorylation of Chk1 mediates the cell cycle arrest^33^. In addition, Chk1 is mainly implicated in the G2 checkpoint arrest. Further, the distribution of the histone variant H2AX (yH2AX)^34^ (Fig. 4A) suggested a significant increase in double-stranded DNA breaks, which is in agreement with the immunoblotting results that indicate the activation of the protein kinase ATM within the DDR signalling cascade. Other studies show possible connections between AHCY and DNA damage; however, the approaches were based on small molecule inhibitors of AHCY^20,35^.

The protein levels of ATM kinase and downstream molecules were also higher, indicating the activation of the response to double-stranded DNA breaks (DSB). In addition to p53 signalling, p27 appeared to activate the regulation of the cell cycle through cyclin signalling, as well as 14-3-3 and GADD45 signalling, which are associated with various DNA stress signals.

We also used another sensitive method, the alkaline comet assay, to gather additional evidence to support the results of the yH2AX screen (Fig. 4B). This method is particularly useful for detecting single strand breaks, alkali-labile sites, double-stranded breaks and several other forms of primary lesions in DNA^36^. Although existing studies focus on the detection of primary DNA damage after exposure to potentially harmful genotoxic compounds^21–24^, the comet test is a valuable resource to study the levels of DNA damage by silencing one particular gene.

The results regarding the primary DNA damage levels in HepG2 cells measured by the alkaline comet assay, and detailed explanations of their statistical significance are shown in Table 1.

**Table 1:** Primary DNA damage in HepG2 cells estimated by the alkaline comet assay. As indicators of DNA damage, tail length, tail intensity, and total area were chosen.

Sample	Without adenosine			With adenosine		
	Tail length (µm)	Tail intensity (DNA%)	Total area	Tail length	Tail intensity (DNA%)	Total area
shCTRL (negative control)	16.03 ± 0.19 15.42 10.42–27.08	0.34 ± 0.03 0.05 0–2.79	667.70 ± 10.28 663.45 180.73–395.49	16.64 ± 0.17* 16.25 11.25–27.50	0.33 ± 0.04 0.04 0–2.51	757.56 ± 10.59* 767.27 211.28–1377.26
shAHCY.1	17.96 ± 0.18^nc,*^ 17.08 13.75–30.83	1.08 ± 0.11^nc^ 0.05 0–14.59	836.69 ± 9.42^nc^,* 788.54 607.47–1638.02	16.46 ± 0.17 15.83 11.67–27.08	2.81 ± 0.30^nc^,* 0.46 0–41.06	703.89 ± 7.97 681.34 278.99–1204.86
shAHCY.2	17.90 ± 0.17^nc^,* 17.08 12.50–26.67	2.03 ± 0.19^nc^ 0.38 0–18.30	860.98 ± 9.65^nc^,* 830.90 483.33–1500.00	16.88 ± 0.17 16.25 11.25–28.75	4.22 ± 0.43^nc^,* 0.31 0–37.70	726.87 ± 8.77 700.61 306.77–1180.21
Positive control	27.33 ± 0.58^#^ 25.00 11.67–60.83	16.14 ± 0.85^#^ 12.16 0–49.56	1042.59 ± 28.13^#^ 949.31 309.20–3360.24	25.80 ± 0.42^#^ 25.00 14.17–52.92	14.67 ± 0.70^#^ 11.36 0–47.52	953.85 ± 18.91^#^ 879.08 313.19–2210.59

shAHCY.1 – cells with silenced AHCY, replica 1; shAHCY.2 – cells with silenced AHCY, replica 2.

Positive control – shCTRL cells exposed *ex vivo* to 50 µM hydrogen peroxide for 10 minutes on ice. For each sample, three replicate slides were prepared.

One hundred independent comet measurements per each slide, i.e. 300 measurements per sample were performed with an image analysis system (Comet Assay IV^TM^, Instem-Perceptive Instruments Ltd. UK) using an epifluorescence microscope (Olympus BX50, Japan) equipped with appropriate filters, under 200x magnification. Results are shown as the mean value ± standard error of the mean (first row), median (second row), and the range of the measured values (min-max; third row).

Statistical significance of data was evaluated using descriptive statistics, ANOVA with *post-hoc* Scheffé’s test (intra-group comparisons) and Mann-Whitney U-test (inter-group comparisons).

The level of statistical significance was set at P < 0.0.05.

The abbreviations above whiskers indicate which samples differ with statistical significance. For ANOVA, the abbreviations are: nc – *vs*. corresponding negative control; # – *vs*. all other samples.

A sign * designates the sample which showed a statistically significant increase of the studied comet parameter compared to its related clone with regard to addition of adenosine.

By selecting distinct comet assay parameters such as tail length, tail intensity, and total area, it was possible to estimate the number of DNA breaks and indicate the difference in DNA damage levels between AHCY-silenced (shAHCY.1, shAHCY.2), and control cells (shCTRL), and increased levels of primary DNA lesions were observed in ACHY silenced cells (Fig. 4A). Additionally, the shCTRL cells (negative control) had a low level of spontaneous DNA damage. This parameter has also been successfully used in the evaluation of comet assay results in previous studies^37–39^. Although the growth with addition of adenosine resulted in a slight increase of tail length and total area in shCTRL cells (Table 1), their DNA damage levels remained very low, and the appearances of their nucleoids did not morphologically differ from the naïve control cells (Fig. 4B).

Two replicas of HepG2 cells with silenced AHCY (shAHCY.1 and shAHCY.2) had comparable levels of primary DNA damage, which both were higher than in shCTRL cells (Table 1). As displayed in Fig. 4B, the appearances of their nucleoids revealed some degrees of damage, visible as the comet-like structures. Adenosine supplementation mostly led to lowering of DNA damage in shAHCY cells, whose damage levels resembled that of shCTRL cells. This was particularly evident from the values obtained for two comet parameters, e.g. tail length and total area (Table 1). As expected, in the positive control samples, we measured significantly increased values of all comet parameters, both in shCTRL cells that have been grown without or with addition of adenosine. Such results prove sensitivity of the applied method for detection of even subtle lesions at DNA level.

### AHCY knockdown affects the HepG2 proteome and transcriptome by activating multiple pathways associated with DNA damage

As the immunoblotting data hinted at a potential mechanism, we widened our understanding of the underlying processes by performing a comparative analysis of the proteome and transcriptome data using Ingenuity Pathway Analysis software (IPA), using log2 (fold change) as a measurement value type for both up- and down-regulated candidates, with a cut-off of 0.5. (Supplementary Tables 1 and 2; mass spectrometry and RNA-Seq data). The primary dataset was derived from a mass spectrometry analysis containing a total of 3,258 (false discovery rate (FDR) <5%) proteins found in the Swiss-Prot (human) database. Data filtering was rigorous, as only proteins occurring in three biological replicas per condition were considered for downstream analysis.

The RNA-Seq metrics showed that the sequence data was high quality, with a Q30 score of 92.38 and an average error rate of 0.38%. An average of 4.66 Gbp of sequence data was generated per sample, providing an average coverage of 57,45 per transcript. The values are based on the human annotation hg19, with a sum base count of all exons in the human genome of 81,105,734 bases (including mitochondrial genome; https://genome.ucsc.edu). Analysis was performed on two biological replicates. The mean average (MA) plot of all detected transcripts is shown in the supplement (Fig. S1).

Primary analysis information for RNA-Seq using Illumina RNAexpress analysis tools showed a read number between 30,460,833 and 33,258,183 for the analysed samples. The percentage of total aligned reads was, on average, 94.6%, with exonic reads ranging from 76.84% to 77.86% of uniquely aligned reads. The abundant reads that align to the mitochondrial or ribosomal sequences ranged from 11.62% to 12.57%. Non-exonic reads accounted for 20.78% to 22.01%, whereas ambiguous reads, e.g., reads overlapping exons from more than one gene, accounted for 1.15% to 1.35%. The annotation gene count was 23,710, with 12,723 genes tested for statistical significance and/or detected for differences in expression levels. The false discovery rate (FDR)-adjusted p-value (q-value) was set to 0.05, yielding 8,253 transcripts. After setting log2 (fold change) to 1, the differentially expressed gene count was 1,418. The list of transcripts is attached in the supplementary data, including information for the mean count, log2 (fold change), standard error log2 (fold change), and q-value. In particular, values for AHCY, as a key transcript of this study, are as follows: log2 (fold change) of −2.59, standard error log2 (fold change) of 0.0678, and q-value of 0. Transcript sequence analysis confirmed the wild-type status of the TP53 gene for the HepG2 cell line.

**Figure 5:**
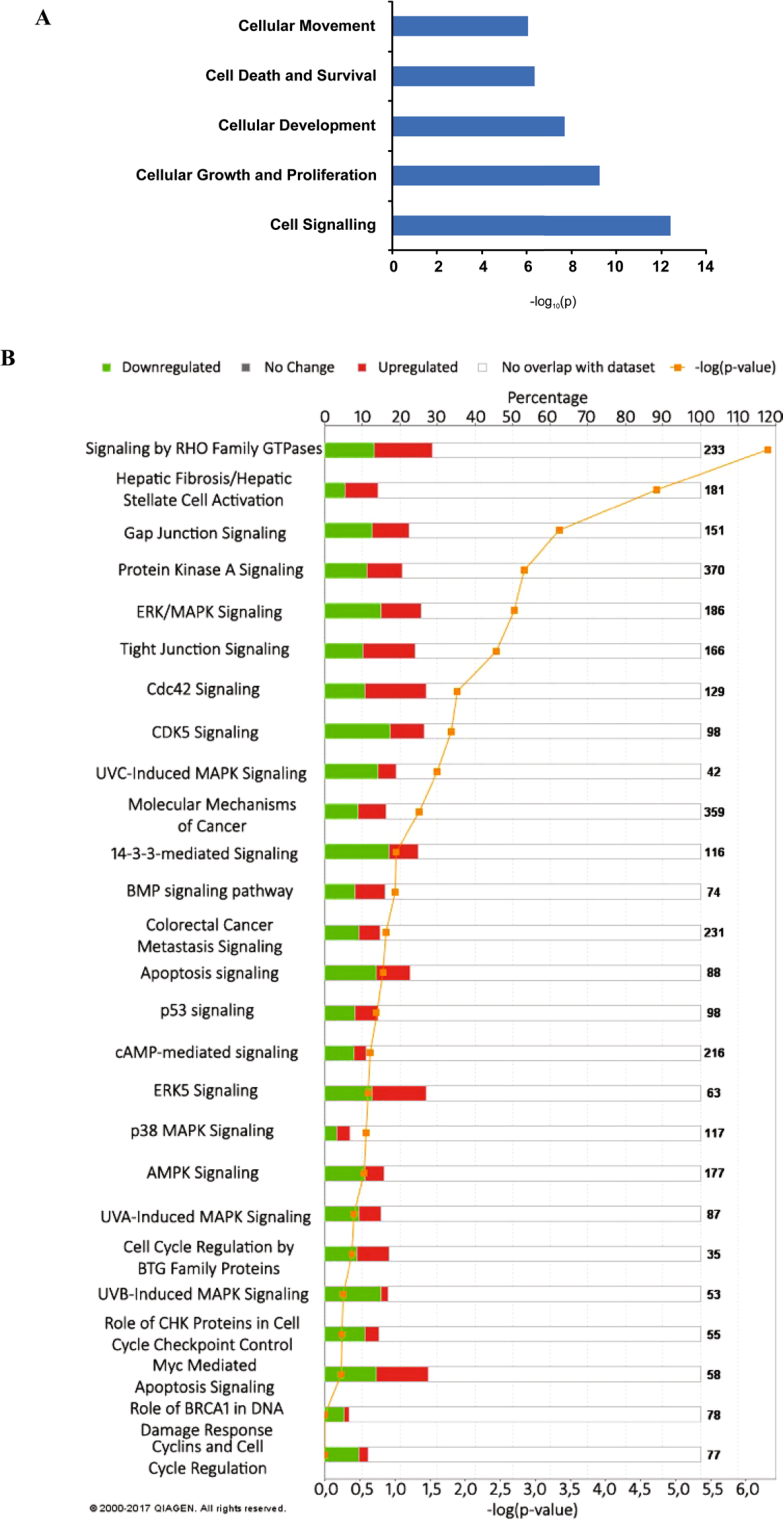
Functional analysis of the omics data performed by IPA software. For both the SILAC and RNA-Seq data, candidates that were imported into the IPA software occurred in all replicates and showed significant up- or down-regulation in AHCY silenced cells compared to the controls. (**A**) The upper section is a rough overview to display common changes in the datasets and the most prominent molecular and cellular functions based on the significance range (p-value). (**B**) The lower section is a graphical representation of the functional categories selected based on the categories that are of the highest relevance to cancer signalling, control of the cell cycle and DNA damage. The p-value is calculated using the right-tailed Fisher’s exact test and was set to less than 0.05 for the upper panel to indicate a statistically significant, non-random association but lowered to 0.00 for graphical representation to evaluate pathways probably associated with the data based on the portion/percentage of the up- and down-regulated or not present molecules involved in the pathway and represented on the bar in red, green or white colour. For each bar, the number of identified up- or down-regulated molecules is shown.

As depicted in Fig. 5, the overlap of the proteome and transcriptome datasets shows that molecular and cellular cancer-related functions are present and functionally divided into separate categories. The integration of both proteome and transcriptome datasets allowed us to address several questions such as (a) how and where pathways intersect and influence each other, (b) are there any less investigated and unexpected cell cycle influencing pathways to be found in the ‘omics’ data, (c) are there any unusual or novel pathways that are highly enriched in our data, d) are there pathways enriched that point to other mechanisms proposed in the literature? Further, we could investigate separately the impact of adenosine depletion on cell metabolism (purine metabolism) versus DNA damage.

The reliability of the analysis can be deduced from the graphic representation of the functional categories, where scoring by the p-value and/or the number of molecules within a given category is a trade-off between a statistically significant, non-random association and the pathways likely associated with the data based on the portion/percentage of molecules involved in the pathway.

Thus, by looking at functional categories affected by AHCY silencing, we found the highest significance for cancer signalling, control of the cell cycle and the DNA damage response (Fig. 5A).

Since HepG2 is a cancerous cell type, it is to be expected that the basic molecular and cellular cancer-related functions are present for the HepG2 datasets. Although functional categories are either very broad and general, such as “molecular mechanisms of cancer” (Fig. 5B), or very specific, such as “hepatic fibrosis/hepatic stellate cell activation”, they serve to assess the reliability of the pathway analysis software (IPA) as an important comparison argument. In parallel, it is expected that molecular and cellular functions may overlap in complex biological systems. This enabled us to distinguish between common cancer-related changes and changes that are AHCY-activity dependent.

The functional categories related to cell cycle control and the DNA damage response (DDR) were in agreement with the results obtained through the above-presented experiments and the observed phenotype. Significant representation of the pathways for ERK/MAPK signalling, 14-3-3-mediated signalling, p53 signalling, Myc-mediated apoptosis signalling, CHK proteins in cell cycle checkpoint control, and cyclins involved in cell cycle regulation was obtained. Other signalling pathways, such as the cAMP and PKA (protein kinase A; cAMP dependent protein kinase) pathways, are well represented in the analysis. cAMP and PKA signalling pathways are often altered in different cancers, thereby providing a source for relevant candidates for targeted cancer therapy and/or diagnostics^40^. PKA is the main intracellular target for cAMP in mammalian cells and is thus affected by the localization and timing of cAMP production. Activated PKA can further act on the RAS/ERK signalling pathway involved in cell cycle progression^41^. Additionally, the cAMP pathway interacts with other intracellular signalling pathways, including the Ras-Raf-Erk pathway^42^. Accordingly, our analysis showed strongly expressed and/or phosphorylated MEK and ERK as well as signalling of their downstream effectors, although Ras and Raf (BRAF) were not activated. Raf is marginally up-regulated, with a log2 (fold change) of 0.583 and a standard error of 0.232 and relatively high q-value of 0.0204, indicating a less significant change in Raf expression, which indicates that cAMP activation is achieved by means other than the upstream Ras/Raf regulators.

Downstream signalling appears to be p53 dependent, most likely through the p53–p21–CDK regulatory module. Other transcriptional targets of p53 are the BTG family of proteins; p53 influences their role in tumour suppression and the DNA damage response, as observed for different cell types^43^. However, other evaluated candidates point to the contribution of additional p53-independent pathways (see Fig. 3), such as p27-activated regulation of the cell cycle through cyclin signalling as well as the activation of 14-3-3 and GADD45 signalling, which is associated with various DNA stress signals.

Additionally, the use of the non-commercial software tool Cytoscape (see methods) on proteomic and transcriptomic datasets nicely complements the data obtained through the IPA tool. In brief, the analysis of significant differences in the protein levels in the primary dataset of 3,258 proteins resulted in 303 proteins (9.3% of all identified proteins), with 161 proteins significantly up-regulated and 142 proteins significantly down-regulated (Supplementary Table 1: SILAC data). Affirmatively, AHCY protein quantity was significantly lower in silenced cells in comparison to the control (B = 1.14E-14). Gene ontology (GO) analysis of the down-regulated proteins in AHCY-silenced HepG2 cells revealed an enrichment of GO categories involved in amino acid, lipid and nucleic acid metabolism. In accordance with the aforementioned results, statistically overrepresented (p < 0.01) GO terms were also proteins involved in DNA repair and nucleotide metabolism (Fig. S2).

In terms of the expression changes that are AHCY-activity dependent, transcriptome profiling showed significant changes in expression levels of members of the methionine pathway (CBS) and the purine synthesis pathway (NT5C, NT5C2, NT5E). Additionally, silenced AHCY activity affected the adenosine-metabolizing enzymes adenosine deaminase (ADA) and adenosine kinase (ADK). Hence, AHCY malfunction should result in significantly decreased levels of both metabolites, and negative feedback should lead to measurable changes in the expression levels of the affected pathways (Supplementary Table 2: RNA-Seq data).

### AHCY silencing effects can be rescued by supplementation with adenosine

To date, adenosine levels have not been determined in AHCY-deficient patients, and we are not aware of any study measuring adenosine in liver cells. Thus, the goals of this study include the comparison of adenosine levels between AHCY-silenced and control samples and the evaluation of the connection between adenosine and the DNA damage response. When comparing the adenosine levels in the control and AHCY-silenced HepG2 cell lysates, significantly decreased adenosine levels were detected in the silenced cells (Fig. 1B). This led us to conduct rescue experiments, wherein we supplemented both AHCY-silenced and control cells with appropriate levels of adenosine and measured the cell cycle parameters and looked for indications of reduced DNA damage. Subsequently, the cell cycle analysis revealed that arrest at the G1/S cell cycle control checkpoint could be reverted for AHCY-silenced cells, which showed a decrease in accumulation in G1 phase to 51.95% and a cell cycle distribution very similar to that of the control cells (Fig. 2C). No changes were observed in the control cells after adenosine supplementation.

DNA damage appeared to be less prominent after adenosine supplementation. This was particularly evident for the values of two comet parameters, which showed significantly increased tail lengths and total areas, respectively (Fig. 4B). Additionally, adenosine supplemented shAHCY-silenced cells showed slightly increased tail intensity, which further reflects the value of the tail moment. We presume that the increased tail intensity could be associated with DNA synthesis and replication due to sufficient amounts of adenosine. It is possible that in cases of intensive DNA replication, the amount of DNA fragments could contribute to the overall level of the damage measured by the alkaline comet assay, which must be studied further.

Considering previous work that showed that hydroxyurea treatment in cancer significantly disturbs dNTP metabolism, in particular, dATP^30^, which is a major contributor of DNA replication, it is likely that a disturbance of adenosine metabolism may significantly impact the dATP levels analogously to hydroxyurea, with implications for DNA replication and the cell cycle. Hydroxyurea acts through inhibition of ribonucleotide reductase (RNR) and thus starves DNA polymerase at the replication forks for dNTPs. It is important to note that RNR activity increases at the G1/S checkpoint to elevate dNTP levels during S phase^44^. Although all four dNTPs are synthesized by RNR, it has been shown that dNTP pools tend to be highly asymmetric in mammalian cells, with increased dATP and dGTP levels^45^. Obviously, disturbances in adenosine metabolism significantly impact dATP levels, with implications for DNA replication and the cell cycle. Moreover, the observed activation of 14-3-3 and GADD45 signalling indicates disturbance of dNTP metabolism particularly given the role of 14-3-3 in stabilizing single-stranded DNA in slow or stalled replication forks as well as in initiating the progression and restart of replication forks that are stalled due to limited concentrations of nucleotides^46^.

**Figure 6:**
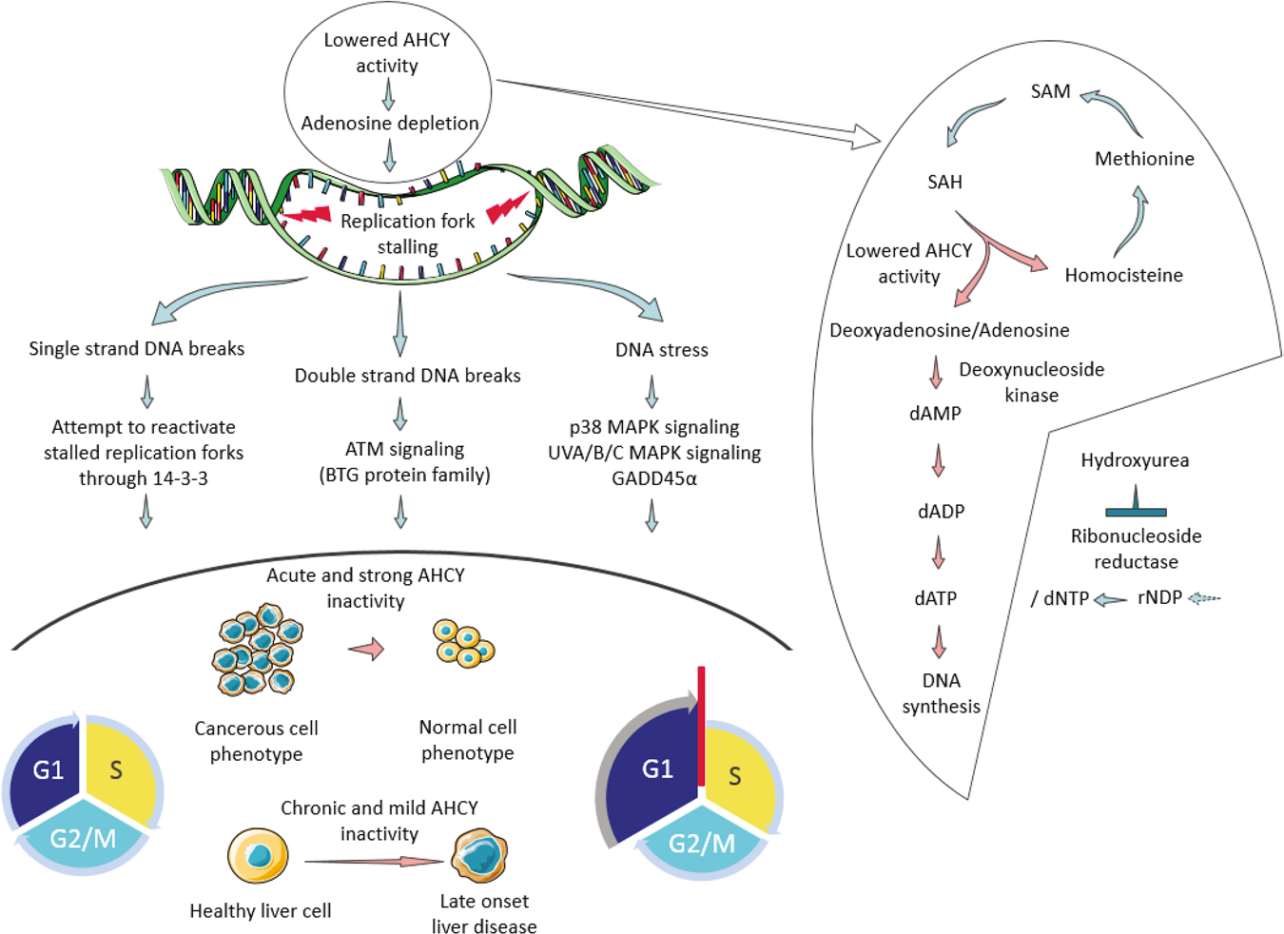
The proposed model that connects AHCY activity, DNA damage and the regulation of the cell cycle through adenosine levels in hepatocellular carcinoma cells. Left: General schematic representation of proposed model: lowered AHCY activity causes adenosine depletion, stalling of replication forks and subsequent DNA damage, which activates various signalling pathways and causes cell cycle arrest in the G1/S checkpoint. Strong and sudden lowering of the AHCY activity causes immediate proliferation changes in cancer cells; however, mild inactivation of AHCY would cause chronic stress for liver cells and thus contribute to adult onset liver disease, such as hepatocellular carcinoma as observed in the latest case of AHCY deficiency. Right: A detailed overview of how adenosine depletion could cause replication fork stalling through misbalance of the dNTP pool due to lower dATP levels, and subsequent impairment of adequate rates of DNA synthesis and progression of the replication forks. Treatment with hydroxyurea, although following another pathway, facilitates a similar and well-described effect through the disturbance of the balance of the total dNTP pool.

In view of the results, we propose a mechanism connecting AHCY activity, DNA damage and the regulation of the cell cycle through adenosine levels (Fig. 6). Overall, lowered AHCY activity cause adenosine depletion, therefore stalling replication forks and subsequent DNA damage that activates various signalling pathways and causes cell cycle arrest at the G1/S checkpoint. A strong and sudden decrease in AHCY activity causes immediate proliferation changes in cancer cells, whereas mild inactivation of AHCY may cause chronic stress for liver cells and thus contribute to adult onset liver disease, such as hepatocellular carcinoma, as observed in the latest case of AHCY deficiency. Adenosine depletion results in lower dATP levels, which causes the replication fork to stall through the misbalance of the dNTP pool, leading to the subsequent impairment of DNA synthesis and progression of replication forks.

## Conclusions

We have presented a mechanism that connects AHCY activity, DNA damage and cell cycle regulation through adenosine levels and draw two major conclusions, which may lead to a refinement of therapeutic procedures for liver cancer as a result of AHCY malfunction and a potentially new approach for targeted cancer therapy based on adenosine depletion. We anticipate a scenario where our results may prompt clinical studies and big data collection focused on adenosine and liver disease.

On one hand, our model may be used to explain adult onset hepatocellular carcinoma in the latest cases of AHCY deficiency^26^. Here, we propose that slight variations of AHCY activity, due to the genetic background of an individual, can be a risk factor for the development of late-onset liver diseases due to chronic DNA stress caused by lowered AHCY activity. Mitigation of the adverse effects of chronic AHCY insufficiency via adenosine supplements may be considered as a dietary precaution.

On the other hand, induced adenosine depletion might be the desired approach in diagnosed cancer to provoke the DNA damage response, cell cycle arrest and apoptosis. We propose that this mechanism might be applied to all cancer types with high expression levels of AHCY and/or are highly dependent on adenosine concentrations for cell survival. The downstream DNA damage response, however, may activate different pathways, causing apoptosis or cell cycle arrest in different checkpoints, depending on the cancer type. Although this mechanism is valid for all other previously reported AHCY-related cancer studies, it has yet to be determined which cancer types are highly sensitive to increases in DNA damage caused by AHCY knockdown and which AHCY inhibitors could be used in advanced and targeted cancer therapy. In particular, considering the fact that liver cancer is the third leading cause of cancer-related deaths worldwide^47,48^, seeking new approaches and initiating new clinical studies is relevant.

Finally, we strongly suggest including the AHCY gene in any mutational screens as a potential risk factor for cancer, as, to our knowledge, it is not yet considered in commercially available cancer panels for personal health genomics.

## Materials and Methods

### Reagents

All chemicals, unless stated, are from Sigma-Aldrich (Merck, Darmstadt, Germany).

### Cell culture and transfection

Cancerous cell lines were obtained from the American Type Culture Collection (ATCC). Mycoplasma detection was performed using a commercially available contamination detection kit by following the manufacturer’s protocol (Thermo Scientific). HepG2 cells were cultured in Dulbecco’s modified eagle’s medium (DMEM) with high glucose content (D5796) and supplemented with 10% foetal bovine serum (FBS; F7524) and 2 mM L-glutamine (SG7513) in a humidified chamber with 5% CO_2_ at 37 °C. For all experiments and assays, the cells were detached using trypsin (T4049) and counted with a Z2 Particle Counter (Beckman Coulter, 6605700). Transfections were performed either by electroporation with a Gene Pulser Xcell™ Total System (BIO-RAD, 1652660) or with Viafect reagent (Promega, E4981) following the manufacturer instructions.

The efficiency of MISSION shRNA clones (Sigma-Aldrich, SHGLY) to silence AHCY expression was evaluated by transfecting HeLa cells that stably express EGFP-AHCY protein. The reduction of EGFP-AHCY fluorescence was assessed by flow cytometry, and the most efficient MISSION clones were used to transfect the HepG2 cell line. Puromycin (Carl Roth GmbH, 0240.1) was added 48 h after transfection to a final concentration of 1 µg/mL to obtain stable shAHCY transformants. The efficiency of AHCY silencing was assessed in whole cell populations or after selecting individual clones using immunoblotting. A stably transformed clone with non-effective (scrambled) AHCY silencing was used as a control for all experiments (shCTRL).

### Determination of SAM and SAH by LC–MS/MS

The liquid chromatography linked to tandem mass spectrometry method for the determination of *S*-adenosylmethionine (SAM) and *S*-adenosylhomocysteine (SAH) in human cells was developed as a modification of a previously published method by Kozich laboratory^49^. Namely, instead of using perchloric acid, we modified procedures in favour of ammonium formate. Stock solutions of SAM and SAH were prepared as 1 mg/mL solutions in cold Milli-Q water (MERCK MILLIPORE, Synergy). Since SAM and [^2^H_3_]-SAM were obtained as *p*-toluene sulfonate salts, exact concentrations were determined by UV-spectroscopy (molar extinction coefficient ε = 15400). Aliquots (0.1 mL) of SAM and SAH stock solutions were diluted together in Milli-Q water to yield concentrations of 10 μg/mL for each substance. [^2^H_3_]-SAM was diluted with 0.1% FA in Milli-Q water. [^13^C_5_]-SAH was obtained from 30.3 μg/mL solution in Milli-Q water. Spiked solutions of labelled SAM and SAH were prepared by dilution of [^2^H_3_]-SAM in Milli-Q water and with addition of [^13^C_5_]-SAH. All standard solutions were stored at −20 °C.

The calibration samples were prepared from SAM and SAH stock solutions in 1 M trifluoroacetic acid (TFA) with addition of isotope labelled internal standard solution (10 μL of spike solution [^2^H_3_]-SAM 7.54 μg/mL and [^13^C_5_]-SAH 2.66 μg/mL). The calibration samples were neutralized with a 5 M solution of ammonium formate just before analysis, and the total volume of each calibration sample was 300 μL. The particular calibration points of SAM and SAH were 11, 28, 56, 111, 333, 833, 1333, and 1666 ng/mL. QC samples were prepared as 333 ng/mL of 1 M TFA/5 M ammonium formate solution SAM and SAH spiked with the same volume of internal standard solution. The calibration curve was obtained by linear regression, and the peak area ratio (analyte/internal standard) was plotted versus the analyte concentration.

Cells were grown in 10-cm plates, and after reaching 80% confluence, they were briefly washed in PBS. Lysis was performed in 1 M TFA on ice. The homogenate was briefly sonicated and cleared after centrifugation at 16 000 × g for 10 min at +4 °C.

A 100-µL aliquot of cellular homogenate was spiked with internal standard solution (10 μL of spike solution [^2^H_3_]-SAM 7.54 μg/mL and [^13^C_5_]-SAH 2.66 μg/mL). Samples were neutralized with 190 µL of 5 M solution of ammonium formate just before analysis, and clear solution was injected to the LC column.

LC–MS/MS analysis was carried out using an Agilent Technologies 1200 series HPLC system equipped with a binary pump, a vacuum membrane degasser, an automated autosampler and an injector interfaced with a 6420 triple quadrupole mass spectrometer with an electrospray ionization source (ESI; Agilent Technologies Inc. Palo Alto, CA, USA). The separation was performed on a Kinetex C18 column (75 × 4.6 mm, 2.6 μm particle size) (Phenomenex, Torrance, USA). Solvents for the analysis were 0.1% FA in water (solvent A) and 0.1% FA in acetonitrile (solvent B). The gradient was applied as follows: 0 min at 100% A, 0–3 min at 100% A, 3–12 min at 100% A-10% A, 12–14 min at 10% A, 14–16 min at 10% A-90% A, and 16–25 min at 100% A. The flow rate was 0.3 mL/min. Between 2.5 and 15 min of each run, eluent was diverted to the ion source, while at the beginning and end of the run, the eluent was diverted to the waste.

The ESI was operated in a positive mode, and samples were detected in the multiple reaction monitoring (MRM) mode, with a dwell time of 200 ms per MRM transition. The desolvation gas temperature was 300 °C, with a flow rate of 8.0 L/min. The capillary voltage was 4.0 kV. The collision gas was nitrogen. The MRM transitions of the precursor to product ion pairs were *m/z* 399.3-250.3 for SAM, *m/z* 402.3-250.3 for [^2^H_3_]-SAM, *m/z* 385.3-136 for SAH and *m/z* 390.3-136.3 for [^13^C_5_]-SAH. The fragmentor voltage for SAM and [^2^H_3_]-SAM was 100 V and the collision energy was set at 10 V. The fragmentor voltage for SAH and [^13^C_5_]-SAH was 100 V and the collision energy was set at 15 V. All data acquisition and processing were performed using Agilent MassHunter software.

### Determination of adenosine in cell samples

Eight million cells were scraped from the cell culture plate in 2 mL of cold Baxter H_2_O and sonicated with a Bioruptor (Diagenode) at +4 °C for 10 cycles at a low setting for 30 sec, with a 30-sec pause in between cycles. Protein concentration in the supernatant obtained by centrifugation of the cell lysates at +4 °C for 15 min at 14,000 rpm was determined using a Qubit 3.0 Fluorometer and Qubit™ Protein Assay Kit (Thermo Fisher Scientific, Q33216, Q33211). Lysates were deproteinised with 2.5 volumes of cold 96% EtOH and then centrifuged at +4 °C for 15 min at 14 000 rpm. Supernatants were evaporated in a Savant DNA110 SpeedVac Concentrator, the pellet was resuspended in Baxter H_2_O, and the deproteinization process was repeated. The final pellet was resuspended in Adenosine Assay buffer and further diluted and measured as recommended by the manufacturer of the Adenosine Assay kit (BioVision). The whole experiment was repeated twice, adenosine was measured in triplicate, and one outlier was removed for statistical analysis.

### Cell cycle analysis by PI staining

For propidium iodide (PI) staining, 800,000 cells per well were seeded 24 h prior to the experiment in 6 well plates, detached and washed with PBS. Cells were fixed overnight at −20 °C after adding ice cold 70% EtOH on a minimum setting vortex. Cells were washed and incubated with 0.1 μg/mL final Ribonuclease A in PBS (RNAse A, R6513) in a 37  °C water bath for 20 min and then with 50 μg/mL final PI in PBS (P4864) for 30 min at RT in the dark. All centrifugation steps were performed at 300 × g for 5 min. Data acquisition and analysis was performed by a FACSCalibur flow cytometer (Becton Dickinson) using CellQuest 3.3 and FlowJo software.

### Cell proliferation assays

Cell proliferation rates were evaluated using two assays and represented as changes in the number of viable cells 48 h after plating 10000 cells per well in 96-well cell culture plates (TPP, 92096).

To prepare the neutral red assay (NRA), a neutral red (N4638) stock solution (4 mg/ml in PBS) was diluted with DMEM (without FBS) to a final concentration of 40 μg/mL and placed at 37 °C overnight. The next day, the cells were washed with PBS, and 100 µL of previously prepared neutral red medium was added to each well and incubated for 4 h in a humidified chamber. Media was removed, cells were washed with PBS, and 150 µL of distain solution (50% EtOH, 1% glacial acetic acid) was added to each well. Neutral red was dissolved by shaking the plates for 10 min (IKA^®^ VORTEX genius shaker 3, setting 3).

The MTT test was performed by applying 20 µL of 5 mg/mL MTT dissolved in DMSO (M5655, D4540) to the growth media to each well. After incubation for 1 h in a humidified chamber, the media was removed, and the cells washed with PBS. Formazan crystals were dissolved by adding 100 µL of DMSO to each well and shaking the plates for 10 min (IKA^®^ VORTEX genius shaker 3, setting 3).

The absorbance (OD, optical density) was measured with a Multiskan EX (Thermo Scientific) microplate reader at 540 nm for NRA or at 570 nm for the MTT test.

### Western blotting

Whole cell proteins were obtained by cell scraping in cold lysis buffer (0.9% NaCl, 2.4% Tris, 0.08% EDTA, 0.01% NP-40) with cOmplete™ Mini Protease Inhibitor Cocktail (11836153001) following sonication on ice (Misonix XL2000 Microson, 5.5 setting). After centrifugation at 14,000 rpm at +4 °C, the protein concentration in the supernatant was determined using a Pierce BCA Protein Assay Kit (Thermo Fisher Scientific, 23225). Proteins were separated on 6–12% sodium dodecyl sulfate-polyacrylamide resolving gels. Immunoblotting was performed as described previously^50^ using the antibodies listed in Table 2.

**Table 2:** Antibodies used for western blotting in alphabetical order

Name	Company	Catalogue number
14-3-3ζ/δ (D7H5)	Cell Signaling Technology	#7413
AHCY	Abcam	ab134966
anti-biotin HRP-linked Ab	Cell Signaling Technology	#7075
ATM	Novus Bio	NB100–309
beta - actin	Abcam	8226
B-Raf (55C6)	Cell Signaling Technology	#9433
cdc2 (POH1)	Cell Signaling Technology	#9116
CDK2	Santa Cruz Biotechnology	sc-163
CDK6 (DCS83)	Cell Signaling Technology	#3136
Chk1	Santa Cruz Biotechnology	sc-8408
cMyc	Santa Cruz Biotechnology	sc-8351
C-Raf	Cell Signaling Technology	#9422
Cyclin A	Santa Cruz Biotechnology	sc-8351
Cyclin B1 (D5C10) XP®	Cell Signaling Technology	#12231
Cyclin D1 (92G2)	Cell Signaling Technology	#2978
Cyclin E (HE12)	Cell Signaling Technology	#4129
FITC Mouse Anti-BrdU (Clone B44)	BD Biosciences	347583
Gadd45α (D17E8)	Cell Signaling Technology	#4632
goat anti-mouse IgG, Alexa Fluor 594	Invitrogen	A-11005
goat anti-mouse IgG, pAb	Enzo	ADI-SAB-100
goat anti-rabbit IgG, pAb	Enzo	ADI-SAB-300-J
MDM2 (SMP14)	Santa Cruz Biotechnology	sc-965
MEK1/2 (L38C12)	Cell Signaling Technology	#4694
p21 waf1/cip1 (DCS60)	Cell Signaling Technology	#2946
p27	Santa Cruz Biotechnology	sc528
p44/42 MAPK (Erk1/2)	Cell Signaling Technology	#9102
p53 (DO-1)	Santa Cruz Biotechnology	sc-126
pChk2 and chk2	Novus Bio	NB100-500
Phospho-B-Raf (Ser445)	Cell Signaling Technology	#2696
phospho-cdc2 (Tyr15) (10A11)	Cell Signaling Technology	#4539
Phospho-C-Raf (Ser259)	Cell Signaling Technology	#9421
phospho-MDM2 (Ser166)	Santa Cruz Biotechnology	sc-293105
Phospho-MEK1/2 (Ser221) (166F8)	Cell Signaling Technology	#2338
phospho-p44/42 MAPK (Erk1/2) (Thr202/Tyr204) (197G2)	Cell Signaling Technology	#4377
phospho-p53 (Ser15)	Santa Cruz Biotechnology	sc-101762
Ras (27H5)	Cell Signaling Technology	#3339
Rb	Abcam	ab24
yH2AX	Abcam	ab11174

### Immunocytochemistry

The yH2AX antibody was used to evaluate the amount of double strand DNA breaks. Approximately 80,000 cells were grown on coverslips for 24 h. The cells were washed with PBS after removing the media and fixed with 2% PFA in PBS for 10 min at RT. After washing two times in PBS, the cell membranes were permeabilized in 0.1% Tween-20 in PBS (P2287) for 10 min at RT and then blocked for 30 min at RT in 0.1% Tween-20 and 1% BSA in PBS. Incubation with phosphor-specific primary γ-H2AX (1 h; Table 2) and secondary Alexa 594-labelled antibody (30 min; Table 2), both diluted in blocking solution, was performed at RT with gentle agitation and protection from light and evaporation with washing 3 × 15 min with PBS after each antibody. Cover slips were mounted on glass slides using Fluoroshield^™^ with DAPI (F6057) to counterstain the cellular nuclei.

### Confocal microscopy and image analysis

Wide-field fluorescence images were obtained using a Leica SP8 X FLIM confocal microscope with an HC PL APO CS2 63 ×/1.40 OIL objective. To image the foci, each image was reproduced in 12 stacks. Images were analysed with ImageJ software to merge the stacks and assess the number of foci representing the areas of DNA damage, as well as to determine the shape and size of the nuclei. Using the ImageJ Measure option, we analysed the area and perimeter as the most informative nuclear morphometric features and the following form factors: circularity (with a value of 1.0 indicating a perfect circle), nuclear roundness (where 1 is in a circular nucleus and less than 1 if the nucleus is elliptic) and AR (area divided by ð/4 × longest axis × shortest axis). The mean was calculated for each nuclear feature, and the obtained data were statistically analysed and compared for the two groups.

### Alkaline comet assay

The assay was performed according to Singh and co-workers^51^ using minor modifications of the protocol. To obtain a single cell suspension, the cultures of both control shCTRL cells and cells with silenced ACHY (shAHCY.1 and shAHCY.2) were handled in the same manner. Cells were first washed with PBS and trypsinized. After two minutes, the complete medium was added, and the cells were gently resuspended. To prepare the slides, aliquots of 10 µL of each cell suspension were mixed with 100 µL of 0.5% low melting point (LMP) agarose and spreader on fully frosted microscopic slides pre-coated with 1% and 0.6% normal melting point (NMP) agarose. The top layer of each microgel consisted of 0.5% LMP agarose. Three microgels per sample were prepared. All slides were coded. To prepare slides for the positive control, shCTRL cells embedded in an agarose microgel were exposed *ex vivo* to 50 µM hydrogen peroxide for 10 minutes on ice.

All slides were transferred into freshly prepared ice-cold lysis buffer (pH 10.0; 100 mM Na_2_EDTA, 2.5 M NaCl, 1% Na lauroylsarcosinate, 10 mM Tris–HCl, 10% dimethyl sulfoxide, and 1% Triton X-100) and lysed overnight at 4 °C. Following lysis, the microgels were arranged in a horizontal electrophoresis chamber, facing the anode. They were first subjected to alkali denaturation in a freshly prepared buffer (pH 13.0; 300 mM NaOH, 1 mM Na_2_EDTA) for 20 min to allow for DNA unwinding and the expression of alkali-labile sites. Both denaturation and electrophoresis (runtime 20 minutes, 25 V, 300 mA) was performed protected from light. After electrophoresis, the microgels were neutralized in three washes with 0.4 M Tris-HCl buffer (pH 7.5) at five-minute intervals and stained with ethidium bromide (20 µg/mL). One well-trained scorer performed all the comet measurements on coded/blinded slides. The level of DNA damage in individual cells was assessed with an image analysis system (Comet Assay IV^TM^, Perceptive Instruments Ltd. UK) using an epifluorescence microscope (Olympus BX50, Japan) equipped with appropriate filters under 200x magnification. A total of 100 randomly selected comets per slide (300 per experimental group) were measured on replicate slides. As indicators of DNA damage, tail length (presented in micrometres), tail intensity (i.e. DNA % in tail), and total area (it represents the overall surface area of the comet) were chosen.

Statistical calculations were performed using the Dell™ Statistica™ 13.2 software (USA). The data were first evaluated using descriptive statistics. Further evaluations were performed using analysis of variance (ANOVA). Before data analysis, to normalize the data distribution, log_10_-transformation was used. Finally, Scheffé’s test^52^ was used for calculations of pair-wise comparisons between control and cells with silenced ACHY, which have been grown without or with the addition of adenosine. To estimate the effect of adenosine on the levels of DNA damage measured in a particular cell population (shCTRL *vs*. shCTRL + A; shAHCY.1 *vs*. shAHCY.1 + A, etc.), the Mann-Whitney^53^ U-test was used. The level of statistical significance was set at P < 0.05.

### SILAC coupled with LC-MS/MS

shAHCY cells were metabolically labelled with the isotopically labelled lysine (^13^C_6_,^15^N_2_-Lys-OH). Stable isotope labelling by amino acids in cell culture (SILAC) was performed by growing cells for ten passages with SILAC-Lys-8-Kit (^13^C_6_,^15^N_2_-Lys-OH, SILANTES, 282986440), following the manufacturer’s recommendations. Prior to the experiment, the incorporation efficiency was verified in 10^6^ cells to be above 98%. Protein lysates were prepared as previously described^54^. Protein quantification was performed using the Pierce BCA Protein Assay Kit (Thermo Fisher Scientific, 23225), and protein extracts from shAHCY (heavy media) and shCTRL (light media) cells were mixed at a 1:1 ratio. Samples were then reduced with dithiothreitol (150 nmols, 1 h, 37 °C) and alkylated in the dark with iodoacetamide (300 nmol, 30 min, 25 °C). The resulting protein extract was diluted 1/3 with 200 mM NH_4_HCO_3_ and digested with 5 µg LysC (Wako, cat # 129-02541) overnight at 37 °C. Finally, the peptide mix was acidified with formic acid and desalted with a homemade Empore C18 column (3M, St. Paul, MN, USA)^55^. Samples were analysed using a LTQ-Orbitrap Velos Pro mass spectrometer (Thermo Fisher Scientific, San Jose, CA, USA) coupled to an EasyLC (Thermo Fisher Scientific (Proxeon), Odense, Denmark). Peptides were loaded directly onto the analytical 25-cm column with an inner diameter of 75 μm and packed with 5-μm C18 particles (Nikkyo Technos Co. Ltd. Japan). Chromatographic gradients started at 97% buffer A and 3% buffer B, with a flow rate of 250 nL/min, and gradually increased to 65% buffer A/35% buffer B over 360 min. After each analysis, the column was washed for 10 min with 10% buffer A/ 90% buffer B. Buffer A: 0.1% formic acid in water. Buffer B: 0.1% formic acid in acetonitrile. The mass spectrometer was operated in positive ionization mode with a nanospray voltage set at 2.2 kV and source temperature at 250 °C. Ultramark 1621 for the FT mass analyser was used for external calibration prior to the analyses. Moreover, an internal calibration was also performed using the background polysiloxane ion signal at m/z 445.1200. The instrument was operated in data-dependent acquisition (DDA) mode and full MS scans with 1 micro scan at a resolution of 60,000 were used over a mass range of m/z 350–2000 with detection in the Orbitrap. Auto gain control (AGC) was set to 10^6^, and dynamic exclusion (60 seconds) and charge state filtering disqualifying singly charged peptides were carried out. In each cycle of DDA analysis, following each survey scan, the top ten most intense ions with multiple charged ions above a threshold ion count of 5,000 were selected for fragmentation at normalized collision energy of 35%. Fragment ion spectra produced via collision-induced dissociation (CID) were acquired in the ion trap, AGC was set to 5e^4^, with an isolation window of 2.0 m/z, activation time of 0.1 ms and maximum injection time of 100 ms. All data were acquired with Xcalibur software v2.2. The MaxQuant software suite (v1.4.0.5) was used for peptide identification and SILAC protein quantitation^56^. The data were searched against an in-house generated database containing all proteins corresponding to *Homo sapiens* in the Swiss-Prot database and the corresponding decoy entries (release July 2013, 20876 entries). A precursor ion mass tolerance of 4.5 ppm at the MS1 level was used, and up to three missed cleavages for trypsin were allowed. The fragment ion mass tolerance was set to 0.5 Da. Oxidation of methionine and protein acetylation at the N-terminus were defined as variable modifications, whereas carbamidomethylation on cysteines was set as a fix modification. Identified proteins have been filtered using a 5% FDR.

Only proteins occurring in all three replicates were considered as an input into Ingenuity Pathway Analysis software (IPA, Ingenuity Systems; see http://www.ingenuity.com).

### Transcriptome profiling – RNA-Seq

Total cell RNA was extracted from 1 × 10^6^ cells using TRIzol^®^ Reagent (Thermo Fisher Scientific, 15596026) following the manufacturer’s instructions. Two different cell passages were used to extract RNA both for shAHCY and shCTRL cells and treated as a biological replicate. RNA quantity was determined using a Qubit 3.0 Fluorometer and Qubit^®^ RNA BR Assay Kit (Thermo Fisher Scientific, Q33216, Q10211). Agilent 2100 Bioanalyzer and Agilent RNA 6000 Nano Kits (Agilent technologies, G2939AA, 5067-1511) were used to assess the sample quality. The NeoPrep Library System and a TruSeq Stranded mRNA Library Prep Kit (Illumina, NP-202–1001) were used to prepare libraries from 90 ng of total RNA. Collected libraries were analysed on a 2100 Bioanalyzer, diluted to 1.4 pM and sequenced on an Illumina NextSeq. 500 System using NextSeq. 500/550 High-Output v2 Kit, with 75 cycles (Illumina, FC-404–2005). Run setup, direct data streaming, demultiplexing and analysis were performed at the BaseSpace Sequence Hub (Illumina) using the RNA Express BaseSpace App with default analysis parameters. Data were used to evaluate p53 status and as input for downstream analysis software.

### Signalling pathway analysis

Ingenuity Pathway Analysis software (IPA, Ingenuity Systems; see http://www.ingenuity.com) or Cytoscape^57^ was used for pathway and interactome analysis.

The IPA Core Analysis was run with the causal network analysis option on the uploaded datasets for either proteome or transcriptome data, providing single datasets. Additional relevant parameters include the measurement value type for SILAC experiment log ratio values or, for transcriptome log2 (fold change), a cut-off range: −0.5–0.5; focus on: both up/down-regulated, and species: human. To perform comparative analysis of the mass spectrometry and RNA-Seq data, the obtained results were compared using the ‘comparison analysis’ option. The p-value was calculated using the right-tailed Fisher’s exact test.

Cytoscape gene ontology (GO) analysis was performed with the Bingo tool using the default parameters^58^. Input data served the primary mass spectrometry data set (3,258 proteins; false discovery rate (FDR) <5%), which was filtered for significant differences using the Perseus v. 1.3.0.4 tool (www.perseus-framework.org) and significance B <0.05 values.

## Electronic supplementary material

Supplementary Information

RNAseq dataset

SILAC dataset
