# Supplementary material for: Knock-down of AHCY and depletion of adenosine induces DNA damage and cell cycle arrest
Source: Sci Rep. 2018 Sep 18;8:14012. doi: 10.1038/s41598-018-32356-8 (PMC6143609; doi:10.1038/s41598-018-32356-8)

Knock-down of AHCY and depletion of adenosine induces DNA damage and cell cycle arrest.

Lucija Belužić[1], Ivana Grbeša[2], Robert Belužić[1], Jong Hoon Park[3], Hyun Kyung Kong[3], Nevenka Kopjar[4], Guadalupe Espadas[5,6], Eduard Sabidó[5,6], Adriana Lepur[1,7], Filip Rokić[1], Ivanka Jerić[8], Lidija Brkljačić[8], Oliver Vugrek[1¶].

[1] Ruđer Bošković Institute, Laboratory for Advanced Genomics, 10000 Zagreb, Croatia

[2] Bar-Ilan University, Cellular and Developmental Biology, Ramat-Gan 5290002, Israel

[3] Sookmyung Women's University, Department of Biological Science, Seoul 140-742, Korea

[4] Institute for Medical Research and Occupational Health, Mutagenesis unit, 10000 Zagreb, Croatia

[5] Proteomics Unit, Centre de Regulació Genòmica (CRG), Barcelona Institute of Science and Technology (BIST), Dr. Aiguader 88, 08003 Barcelona, Barcelona
[6] Universitat Pompeu Fabra (UPF), Dr. Aiguader 88, 08003 Barcelona, Barcelona

[7] BIOCenter, Microbiology Laboratory 10000 Zagreb, Croatia

[8] Ruđer Bošković Institute, Division of Organic Chemistry and Biochemistry, 10000 Zagreb, Croatia

Supplementary

Figure S1: MA plot showing a typical pattern of normalized mean read count / log fold change distribution. q-value (multiple testing-adjusted p-value) is used as a measure of statistical significance for differential expression, and transcripts with normalized mean count of less than 10 are excluded from the plot. Total of 8,253 transcripts are considered to be differentially expressed based on q-value<0.05; different colours indicate the degree of significance, and transcripts with lowest q-values (<0.001) are shown in red.

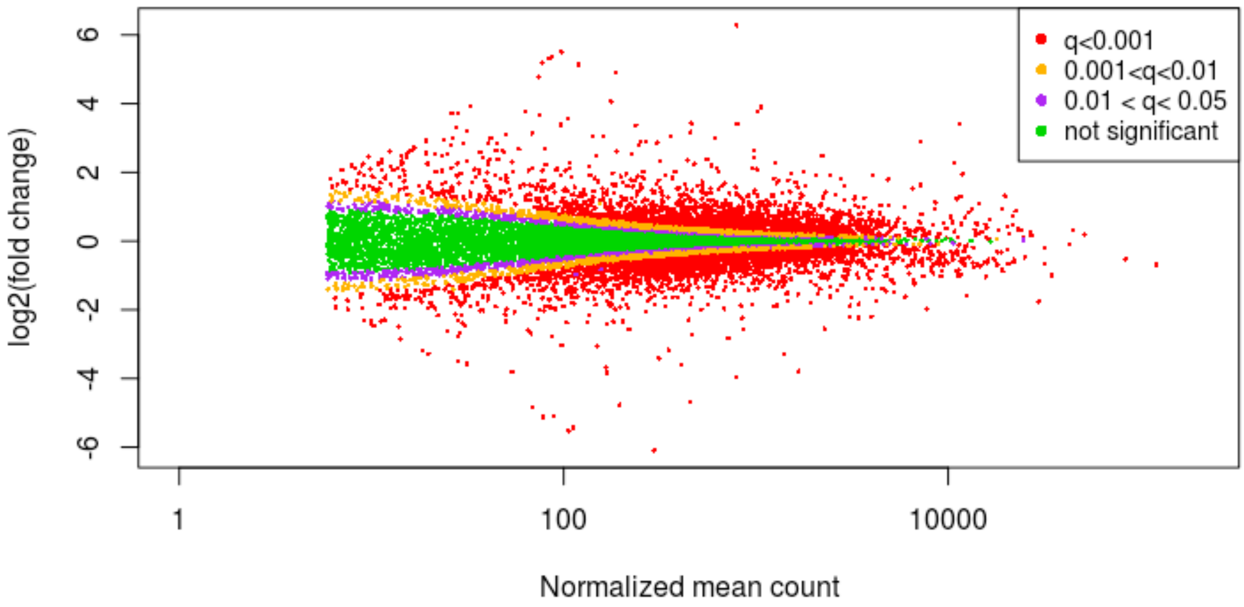

q<0.001
0.001<q<0.01
0.01 < q< 0.05
not significant
log2(fold change)
6
4
2
0
-2
-4
-6
1
100
10000
Normalized mean count

Figure S2: To determine which Gene Ontology (GO) categories are statistically overrepresented we used BiNGO application (Maere et al., 2005) for Cytoscape v.3.5.1. software platform.

The analysis was performed using the Hyper-geometric Test with Benjamini-Hochberg False Discovery Rate (FDR) correction (corrected p-values are shown in figure legend). The overrepresented GO categories (at FDR < 0.05) were the ones for various metabolic processes, nucleotide metabolism, cellular localization/migration, and DNA repair.

A) Cytoscape analysis using only data of significantly up-regulated proteins after AHCY silencing of HepG2 (p < 0.01). Visible is representation of proteins involved in metabolism and DNA repair. The primary dataset derived from mass spectrometry analysis contained a total of 3,258 (false discovery rate (FDR) < 5%) proteins found in the SwissProt (human) database.

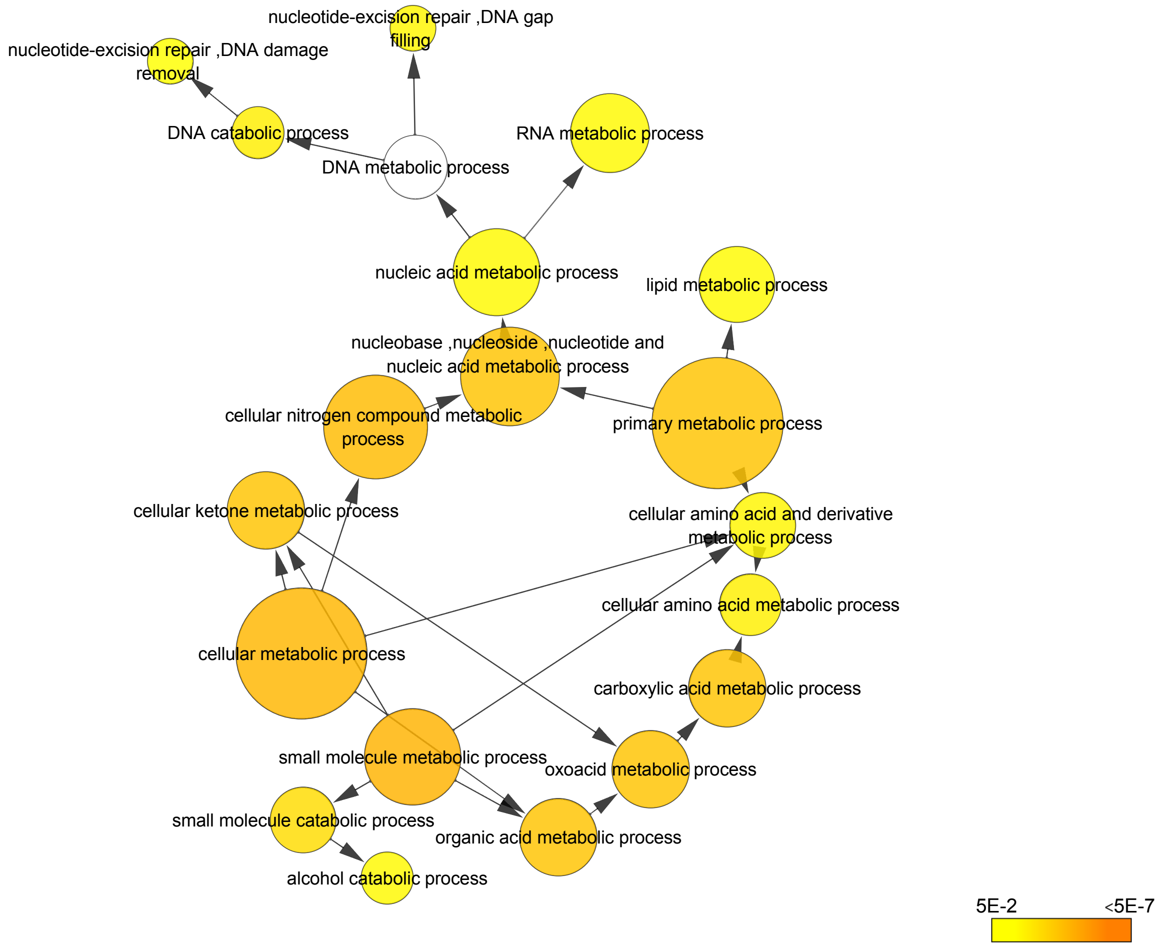

Figure S2: B) Cytoscape gene ontology analysis for all up- and down-regulated genes retrieved from SILAC mass spectrometry data (logFC≥0.5 and ≤0.5, q ≤0.05).

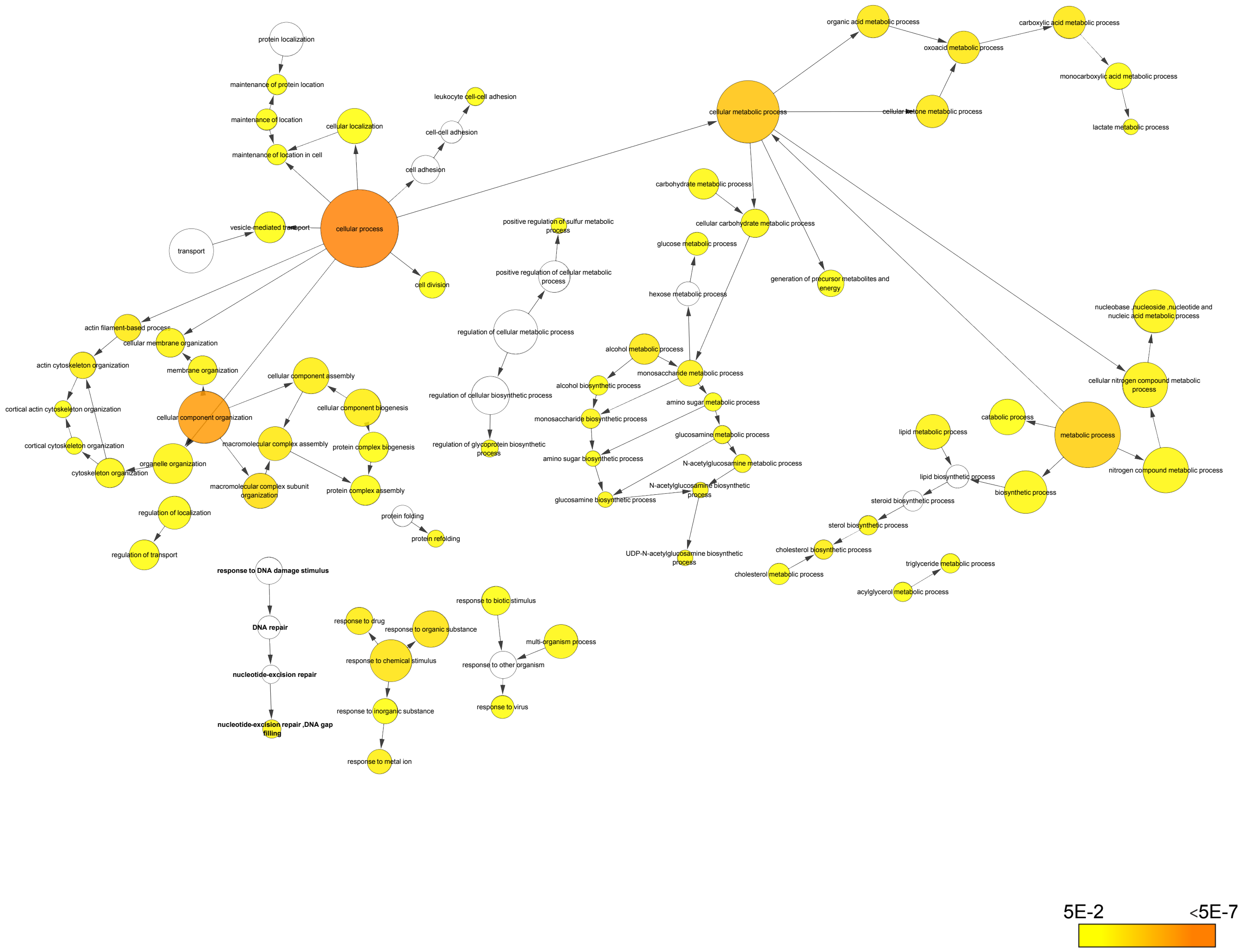

Figure S2: C) Cytoscape gene ontology analysis for up-regulated genes retrieved from RNA-Seq data (logFC≥0.5, q ≤0.05).

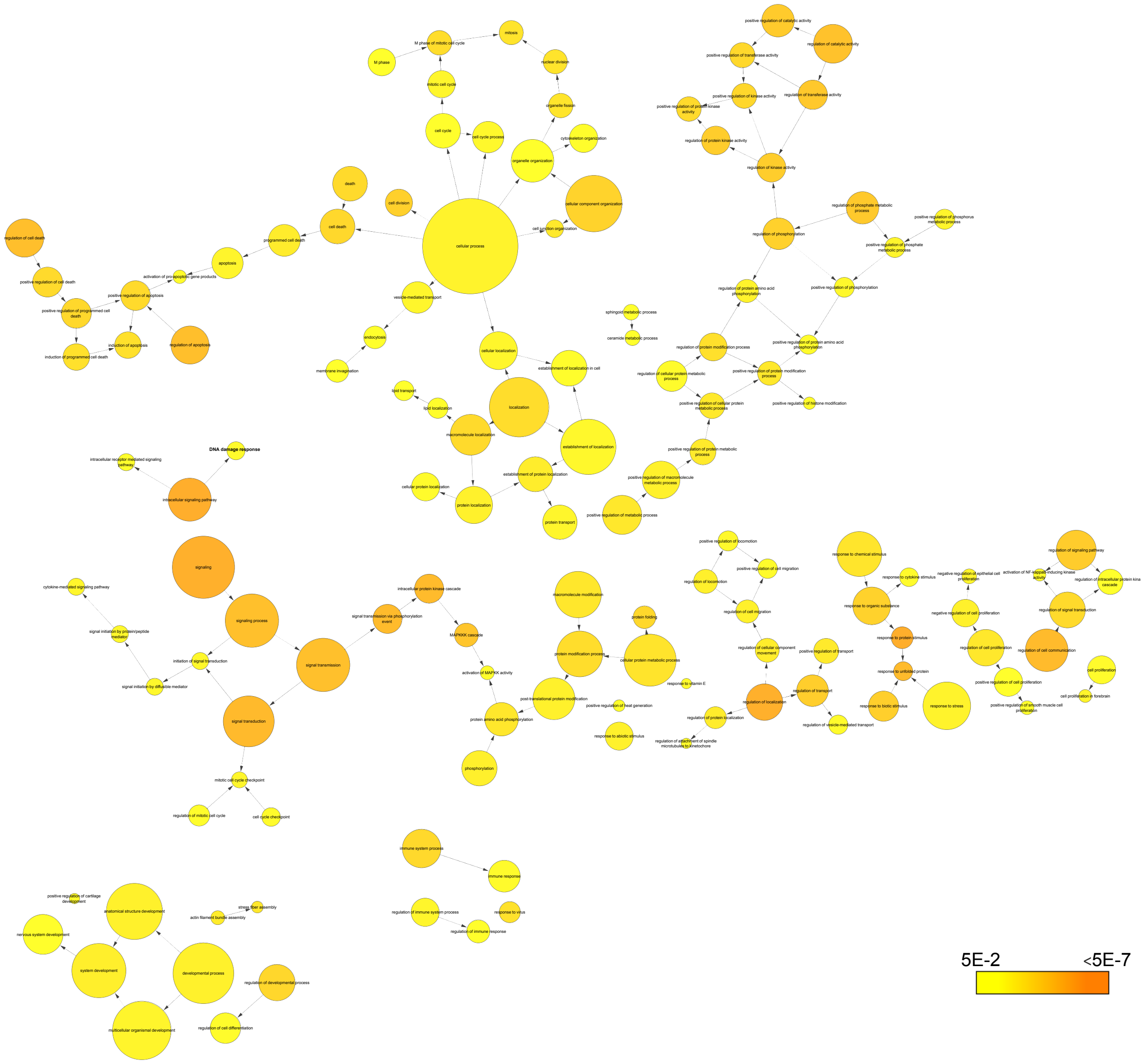

Figure S2: D) Cytoscape gene ontology analysis for down-regulated genes retrieved from RNA-Seq data (logFC ≤0.5, q ≤0.05).

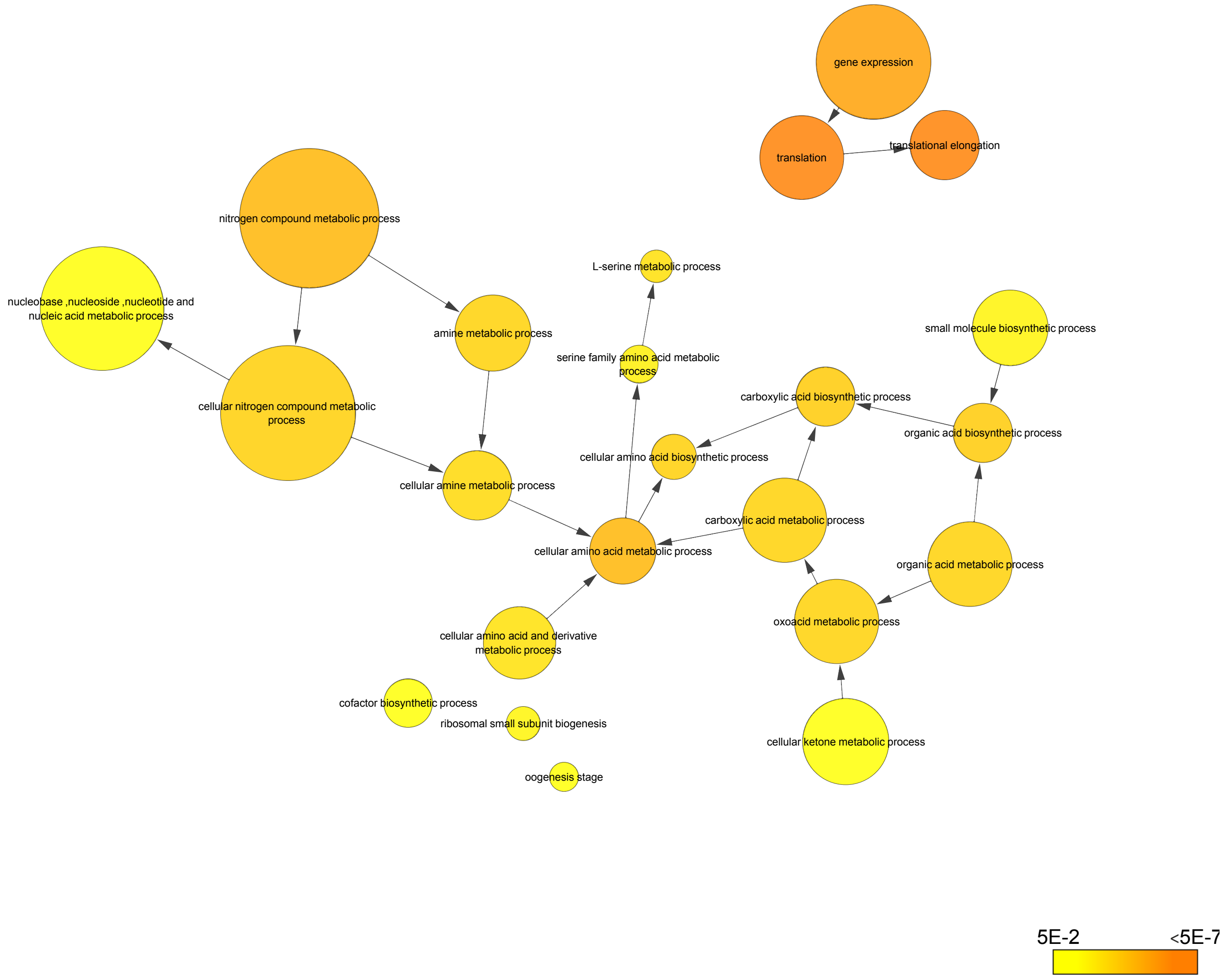

Figure S2: E) Cytoscape gene ontology analysis for all up- and down-regulated genes retrieved from RNA-Seq data (logFC≥0.5 and ≤0.5, q ≤0.05).

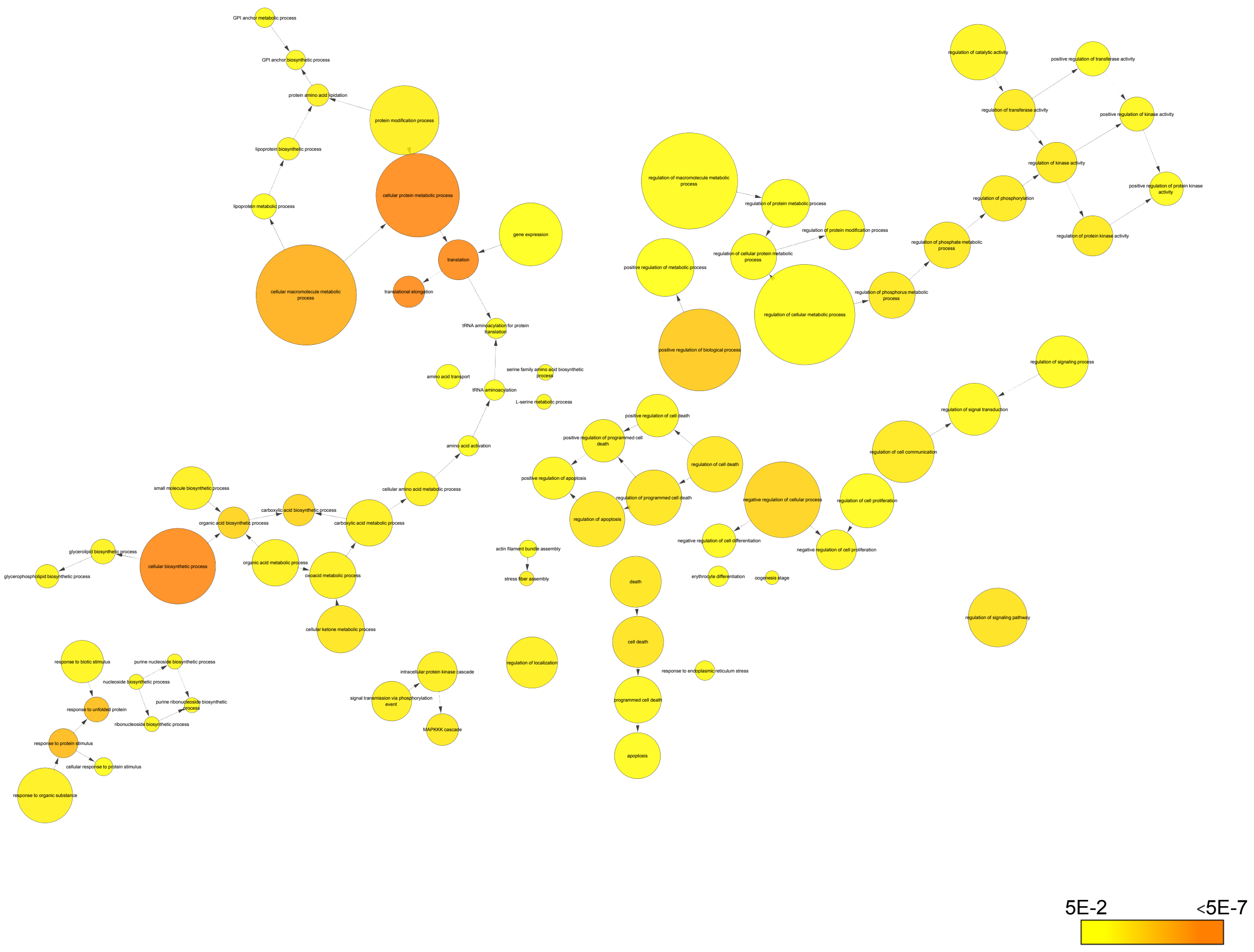

Supplement: Supplementary file 1 — Supplementary Information [file 41598_2018_32356_MOESM1_ESM.pdf]
